# The Impacts of Illegal Toxic Waste Dumping on Children’s Health: A Review and Case Study from Pasir Gudang, Malaysia

**DOI:** 10.3390/ijerph18052221

**Published:** 2021-02-24

**Authors:** Mohd Faiz Ibrahim, Rozita Hod, Haidar Rizal Toha, Azmawati Mohammed Nawi, Idayu Badilla Idris, Hanizah Mohd Yusoff, Mazrura Sahani

**Affiliations:** 1 Department of Community Health, Faculty of Medicine, Universiti Kebangsaan Malaysia, Jalan Yaacob Latif, Bandar Tun Razak, Cheras, Kuala Lumpur 56000, Malaysia; faizibrahim01@gmail.com (M.F.I.); azmawati@ppukm.ukm.edu.my (A.M.N.); idayubadilla.idris@gmail.com (I.B.I.); drhanizah@gmail.com (H.M.Y.); 2 Malaysian Society for Environmental Epidemiology (MySEE), No. 41, Jalan Persiaran Taman Melati, Taman Melati, Setapak, Kuala Lumpur 53100, Malaysia; hrt-warhead@hotmail.com (H.R.T.); mazrura@ukm.edu.my (M.S.); 3 Public Health Division, Johor State Health Department, Jalan Persiaran Permai, Johor Bahru 81200, Malaysia; 4 Center for Toxicology and Health Risk Studies, Faculty of Health Sciences, Universiti Kebangsaan Malaysia, Jalan Raja Muda Abdul Aziz, Kuala Lumpur 50300, Malaysia

**Keywords:** environmental exposure, environmental pollution, chemical pollutants, vulnerable population

## Abstract

Poor management of hazardous waste can lead to environmental pollution, injuries, and adverse health risks. Children’s exposure to hazardous waste may cause serious acute and chronic health problems due to their higher vulnerability to the toxic effects of chemicals. This study examines an incident of illegal chemical dumping in Pasir Gudang, Malaysia and its potential health impacts on children. The study introduced a risk assessment of possible health-related effects due to chemical contamination based on a real case scenario where quantification of the contamination was not feasible. A literature review and spatial analysis were used as research methods. On 6th March 2019, tons of hazardous waste were illegally disposed into Kim Kim River, Pasir Gudang, Malaysia. They were identified as benzene, acrolein, acrylonitrile, hydrogen chloride, methane, toluene, xylene, ethylbenzene, and d-limonene. As a result, 975 students in the vicinity developed signs and symptoms of respiratory disease due to the chemical poisoning. The findings of this study indicate that more effective policies and preventive actions are urgently needed to protect human health, especially children from improper hazardous waste management.

## 1. Introduction

Poor hazardous waste management has become a serious issue in developing countries [1,2,3]. Most cities in those nations are rapidly industrializing, leading to a greater generation of hazardous waste [4,5]. There are inadequacies in the implementation of regulations related to hazardous waste treatment and final disposal of hazardous materials, which have posed significant risks to the environment, ecology, and human health [6,7,8]. The impacts of illegal waste dumping vary depending on the scale, source, and waste characteristics [9]. Among the factors that have influenced the hazardous waste management practice in developing countries are disposal technological capability, supervision and enforcement, waste management policy, and compliance from the corporate companies [10,11].

Waste management has been a long-standing issue in Malaysia [12,13]. One of the main challenges in waste management is managing the scheduled waste that involves collection, transportation, treatments, and disposal of the scheduled waste [14]. The Environmental Quality (Scheduled Wastes) Regulations 2005 (amendment 1989) defined scheduled wastes as any waste falling within the categories of waste listed in the First Schedule, which includes 77 scheduled waste code categories [15]. Some categories of waste in the Schedule are classified as hazardous waste due to their toxicity and hazardous characteristics [16]. Since 1995, Kualiti Alam Sdn Bhd, a privately owned company, has been given exclusive rights and responsibilities to integrate the collection, treatments, and disposal of scheduled waste in Malaysia [17]. Despite having regulations and establishment of the hazardous waste management policies, the event of illegal dumping of hazardous waste has occurred sporadically in the country [18,19].

The Department of Environment (DOE) under The Ministry of Environmental and Water is the responsible agency to ensure the industry’s compliance with the environmental protection rules and regulations. However, several obstacles present challenges to the implementation of scheduled waste regulations. A study by Khoo et al. [20] found that manufacturing industries in the state of Malacca generally had poor compliance to all the three occupational and environmental health-related laws in Malaysia, which are the Occupational Safety and Health Act (OSHA) 1994, Environmental Quality Act 1974 (Act 127) and Guided Self-Regulation Environmental Mainstream Tools (EMT). In 2004, metal hydroxide sludge from Taiwan was illegally imported and stored at Simpang Renggam, Johor [21]. Another similar incident was in Segamat, Johor that caused the evacuation of 300 villagers from their homes after 300 tons of toxic waste was buried at an illegal dumpsite, emitting ammonia fumes [22]. In 2019, Malaysia was shocked by an incident of chemical poisoning in Pasir Gudang that caused thousands of people to feel sick after being exposed to a mixture of chemicals illegally dumped into the Kim Kim River, Pasir Gudang, Malaysia [23,24,25,26]. Although there are many studies involving incidents of illegal waste disposal that have been documented [18,19,20,25,26], to date, no studies have examined the health impact of improper disposal of hazardous waste on children in Malaysia.

The objective of this study is to identify and estimate the potential health risks to children associated with poor hazardous waste management based on a case study in Pasir Gudang, Malaysia. Health risk assessment of a chemical incident on children is generally conducted by evaluation and quantification of contaminants for children [27,28,29]. However, this approach is not always possible in a real crisis or disaster [30]. Hence, this study introduced a risk assessment based on a real case scenario relating to environmental contamination of chemical compounds and the analysis of its possible health-related effects to children. The findings will serve as an important reference to researchers and policy makers in improving policies on environmental protection and public health.

## 2. Methods

### 2.1. Study Area

Malaysia is experiencing a rapid economy development and continuous improvement in living standards. Malaysia’s economy is the most competitive economy among the developing countries in Asia. It ranked 25th out of 140 economies in the global index [31]. The growth of the economy and population is posing a burden on the environment due to greenhouse gases emission, environmental degradation, and waste generation, just to name a few. According to the UN environment report [32], Malaysia generated about 1,600,000 tons of all classes of hazardous waste per year, the third among Association Of Southeast Asian Nations (ASEAN) countries behind Thailand and Philippines, which generated about 3,300,000 and 1,700,000 tons per year, respectively. The manufacturing sector in Malaysia is the leading sector generating toxic and hazardous waste [33]. As waste generation is expected to rise with the population growth and economic development, Malaysia is likely to experience an increase in hazardous waste generation.

**Figure 1:**
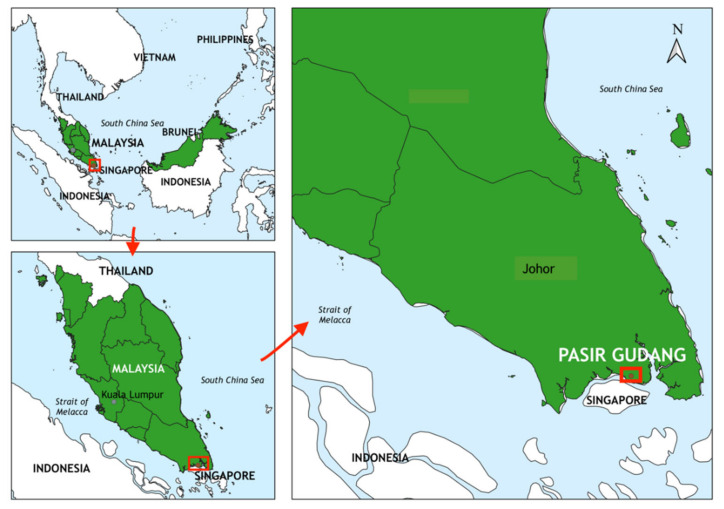
Location of Pasir Gudang.

Pasir Gudang is a significant industrial and port city in Malaysia [34]. It is located at the southwestern part of Johor Bahru district, Johor, Malaysia (Figure 1). Established in 1918, Pasir Gudang was formerly a fishing village that was then transformed into a rubber estate during the British rule in Malaya. Subsequently, the area was further developed by the Johor State Government into residential and industrial areas [35]. In the present day, Pasir Gudang has developed into a port and industrial town. The main economic activities include transportation and logistics, palm oil storage, manufacturing of electronic goods, and other heavy industries such as petrochemicals and shipbuilding [36,37,38]. It has two ports, namely Johor Port and Tanjung Langsat Port, which are among the busiest ports in Malaysia.

Residents of Pasir Gudang are prone to environmental pollution due to its rapid development and industrialization. Prior to the incident of illegal toxic waste in 2019, the environment in Pasir Gudang specifically and areas around the Johor Straits in general were exposed to pollutants resulting from anthropogenic activities [26,39]. The 15 km-long Kim Kim River, running from the Masai sub-district and flowing southward in Pasir Gudang into the Straits of Johor, is considered one of the most polluted rivers in Johor Bahru [40,41]. Keshavarzifard et al. [40] discovered a high level concentration of total polycyclic aromatic hydrocarbons (PAHs) on the surface of the Kim Kim River, which may increase the risk of developing cancer.

### 2.2. Data Source

Information relating to the number of children affected, types of chemical agents involved, sequence of incidents, and the name of affected schools was collected through reports from the official website of the Ministry of Health (MOH), Ministry of Education (MOE) and Department of Environment (DOE). In addition, news from local and international agencies were also included. The key words used were “Kim Kim River,” “illegal dumping,” “pollution,” and “toxic waste.” Only news reported in English and Malay language were included. News related to the event of illegal toxic waste dumping in Pasir Gudang from 6 March 2019 to 20 March 2019 were compiled and arranged into a narrative. Data were collected within the period from September 2020 to November 2020. The location of the dumping site and schools affected was recorded and mapped using QGIS version 3.10.

### 2.3. Health Risk Assessment

**Figure 2:**
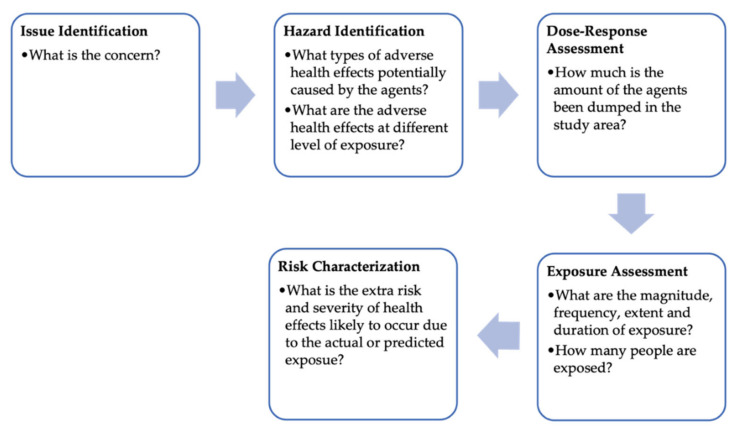
Framework of health risk assessment [29].

The health risk assessment framework for this study (Figure 2) was adopted from the United State Environmental Protection Agency (EPA)’s methodology [42]. In this study, the issue identification was based on the case study of illegal toxic waste dumping in Pasir Gudang, Malaysia. For the hazard identification step, the chemical agents found at the dumping site were assessed to determine its potential to cause negative health effects based on toxicological, epidemiological, in vitro, and mechanistic studies. Evidence on associated exposure-related illnesses was outlined and any data on the risk of acute and chronic health outcomes were considered. The exposure assessment was conducted by identifying the following components:

- exposed population
- exposure pathways
- source of exposure
- time of exposure
- number of people affected

In the risk characterization step, information from the hazard identification and exposure assessments were used to estimate the severity of health effects likely to occur due to the incident. In order to determine the extent of the effects of those chemicals to the environment and human health, the environmental fate of the chemicals was evaluated.

### 2.4. Wireless Information System for Emergency Responders (WISER) Protective Distance and Hybrid Single-Particle Lagrangian Integrated Trajectory (HYSPLIT) Backward Trajectory

In this study, the Wireless Information System for Emergency Responders (WISER) protective distance was implemented to identify the areas likely to be affected in the first 30 min after the chemical substance was released. The WISER was developed by the United States National Library of Medicine to assist emergency responders in hazardous material incidents [43]. This method has been used by the responders during the incident of Kim Kim River [23]. Acrolein and acrylonitrile were selected as the substances. North east wind direction, large spill details, and daytime were chosen. The north east wind direction was chosen because of the predominant wind direction during the northeast monsoon in Malaysia. Malaysia experiences two monsoon seasons, the southwest monsoon (June–September) and the northeast monsoon (December–March). The southwest monsoon wind pattern brings more dry weather to the Peninsula of Malaysia. The northeast monsoon, however, carries strong northeast winds passing over the Peninsula of Malaysia, bringing more rainfall to the east coast of the Peninsular.

A Hybrid Single-Particle Lagrangian Integrated Trajectory Model (HYSPLIT) back trajectory simulation was used for determining the wind direction. Back trajectories started from Pasir Putih Secondary School (1.474° N, 103.937° E), Taman Pasir Putih Primary School (1.475° N, 103.937° E), Tanjung Puteri Resort Primary School (1.454° N, 103.932° E), and Nusa Damai Secondary School (1.498° N, 103.899° E) on the 7th, 11th, 12th, and 13th March 2019, respectively. The trajectory start time was chosen at 12:00 (UTC) to represent 08:00 (local time). A release height of 50 and 100 m above the ground level and 24 h back trajectories with a 1 h interval was chosen. This study assumed that the wind speed is constant, and the pollutant is a passive substance and remains connected throughout its motion without being distorted by the turbulent flow.

## 3. Results

### 3.1. Case Study

#### 3.1.1. First Wave

**Figure 3:**
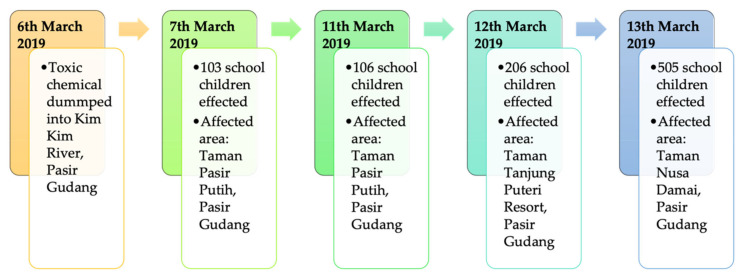
Chronology of chemical pollution in Kim Kim River, Pasir Gudang.

On the morning of 7 March 2019, several students from the Pasir Putih Primary School and Pasir Putih Secondary School experienced a difficulty in breathing, cough, nausea, vomiting, and eye and throat irritation after inhaling an unpleasant odor in their school compound [44]. Some of the residents in Pasir Putih residential area also noticed a strong foul smell in the early morning. A response team from Pasir Gudang health clinic rushed to the schools after receiving emergency calls from both schools. Due to the high numbers of victims, the response team was required to call for additional back-up teams from Masai Health Clinic and Sultan Ismail Hospital, respectively. The Johor State Health Department confirmed that a total of 35 people, mostly students, were sent to Sultan Ismail Hospital for poisoning symptoms [45]. Out of the 35 people, 21 people were admitted for inhalation injury, including 3 in an Intensive Care Unit (ICU), 5 under observation at the emergency unit, and the rest treated as outpatients [45]. Subsequently, the two schools were closed on the same day of the incident for decontamination and the number of school children taken to health facilities rose to 103 people (Figure 3) [46].

#### 3.1.2. Second Wave

On 11 March 2019, a second wave of chemical poisoning took place a few hours after the two schools reopened [46]. More than 100 students were reported to have breathing difficulties and were sent to the nearest clinics and hospitals. As the number of victims among school children grew to more than 500 people on 14 March 2019, the education ministry ordered all 111 schools in Pasir Gudang to be closed [47,48]. A medical base was set-up at the Pasir Gudang Indoor Stadium to receive chemical poisoning-related illnesses and to coordinate the medical response by the Ministry of Health, private agencies and non-governmental organizations (NGOs). Until 20 March, a total of 5039 students and residents were treated due to the exposure of hazardous chemical waste dumped into Kim Kim River [25].

#### 3.1.3. Response and Mitigation

**Figure 4:**
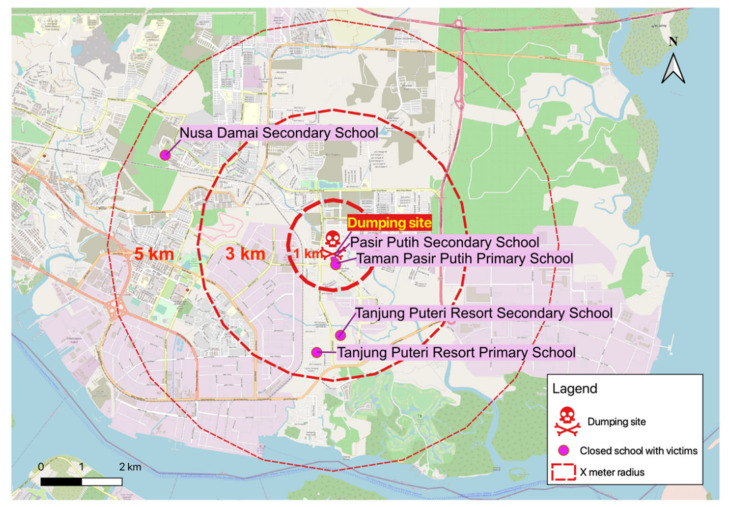
Location of the dumping site and schools affected.

According to the National Police Chief, the incident of Kim Kim River pollution in March was due to illegal dumping of scheduled waste in the river [49]. A tanker lorry from outside Pasir Gudang dumped 2.43 tons of chemical waste that released benzene, acrolein, acrylonitrile, hydrogen chloride, methane, toluene, xylene, ethylbenzene, and d-limonene [50]. The illegal dumping of toxic waste into the Kim Kim River occurred near Kota Masai on the night of 6 March 2019 (Figure 4) [51,52,53]. The contamination of the Kim Kim River had cost huge economic losses. The cleaning operations involving a 1.5 km stretch of the river cost approximately RM10 million [54]. Apart from the Kim Kim River clean-up efforts, the government had also established a scientific committee that served to identify the causes and environmental fate of the pollutants, and their impact on the environment and human health. The committee consists of academicians from a local university and private companies, as well as relevant government agencies including the Department of Environment, Malaysian Space Agency, Malaysian Remote Sensing Agency, Meteorology Department, Department of Health, and Department of Chemistry [55].

The incident received nationwide media coverage, as well as the attention from politicians and royal family [56,57,58]. The Johor State Government approved an emergency aid of RM6.4 million for cleaning works and compensation to the children who were admitted due to the exposure of the hazardous chemicals [59]. Be that as it may, the responses and actions of the government in dealing with this incident have also been criticized for the lack of transparency of the information and the accuracy of the reports that caused confusion among the public [60,61,62].

### 3.2. Health Risk Assessment Results

#### 3.2.1. Issue Identification

The illegal toxic waste dumping on 6 March 2019 in Pasir Gudang has caused public concern over the short- and long-term health effects as it had affected more than 5000 people, mainly children, with breathing difficulties, nausea, vomiting, and irritations to the eye and throat after being exposed to toxic gases at their school. Most of the children experienced respiratory difficulty after inhaling the foul odor circulating within their school. A total of 111 schools were closed to protect the children from any negative health effects.

#### 3.2.2. Hazard Identification and Dose–Response Assessment

A total of 2.43 tonnes of hazardous waste were dumped into Kim Kim River. The chemical waste was brought into Pasir Gudang by a lorry from an unknown location and dumped into the river on the night of 6 March 2019. The dumping site is located 500 m away from Taman Pasir Putih Primary School and Pasir Putih Secondary School. The hazardous wastes have been identified as benzene, acrolein, acrylonitrile, hydrogen chloride, methane, toluene, xylene, ethylbenzene, and d-limonene. Exposure to any of these chemicals may cause harmful health effects. The details of hazard and dose–response assessment is summarized in Table 1.

#### 3.2.3. Environmental Exposure

A total of 975 school children aged 6 to 17 years old were affected in the Kim Kim River incident. The school children were students from five public schools located within a 5 km radius from the dumping site. Among the symptoms reported were difficulties in breathing, cough, eye and throat irritation, nausea, and vomiting. The most possible route of exposure is by inhalation due to upper and lower respiratory tract symptoms among victims. It is difficult to assess which pollutants have actually caused the adverse health effect to the children because the level of chemical concentrations was not available publicly. However, based on chemicals characteristics in Table 1, there is a possibility that the cause of respiratory symptoms among the children was due to exposure to acrolein and acrylonitrile.

#### 3.2.4. Risk Characterization

Children have been identified as the most sensitive receptor in the Kim Kim River incident. The toxic waste was disposed 500 m from the schools, therefore placing children at the highest risk of exposure to the contaminants. Furthermore, the disposal had occurred at dawn, causing the toxic waste to evaporate in the morning while the children were in their classroom. Even if the dose of exposure to children is the same as to adults, children are more susceptible to the effects of pollutants [65,66], putting them at higher risk of various health effects. Children are more vulnerable because of their biological and sociological factors [67,68,69]. The respiratory rate of children is higher than adults, hence resulting in an increased amount of pollutants being inhaled as compared to adults [64]. In addition, their lungs and brains are not fully developed, thus making them more susceptible to these pollutants. Crowded and enclosed areas such as classrooms also played significant roles that increased the risk of these children to be exposed to more pollutants compared to places like residential homes [70]. Aside from the above, children breath-in a higher concentration of pollutants due to their height, which is shorter compared to an ordinary adult, as some heavier pollutants are concentrated more and closer to the ground level [71].

#### 3.2.5. Environmental Fate

The environmental fate is determined by events following the release of chemicals into the environment. Among the most toxic chemicals in the incident of Kim Kim River are acrolein and acrylonitrile. Both acrolein and acrylonitrile vapor are heavier than air; hence, inhalation of acrolein and acrylonitrile may result in respiratory distress especially in enclosed, poorly ventilated, or low-lying areas [72,73]. Acrolein is relatively unstable in the atmosphere and its atmospheric half-life is estimated to be about 15 to 20 h [74]. In the troposphere, acrolein reacts with hydroxyl radicals and ozone that then produces carbon monoxide and formaldehyde. High exposure to carbon monoxide and formaldehyde are also harmful and can cause negative health effects. The level of airborne concentration of formaldehyde that may cause irreversible or serious long-lasting adverse health effects is 14 ppm within 10 to 30 min of exposure, which is 30 to 140 times more potent than the concentration of acrolein [64]. Like acrolein, air-emitted acrylonitrile reacts mainly in the troposphere with photochemically produced hydroxyl radicals. Its atmospheric half-life is estimated to be about 5 to 50 h [75]. The reaction of acrylonitrile with hydroxyl radicals forms formaldehyde and, to a lesser degree, formyl cyanide that would react with water to produce hydrogen cyanide, which is unlikely to cause health effects in a low concentration [76].

### 3.3. WISER Protective Distance and Backward Wind Trajectories

**Figure 5:**
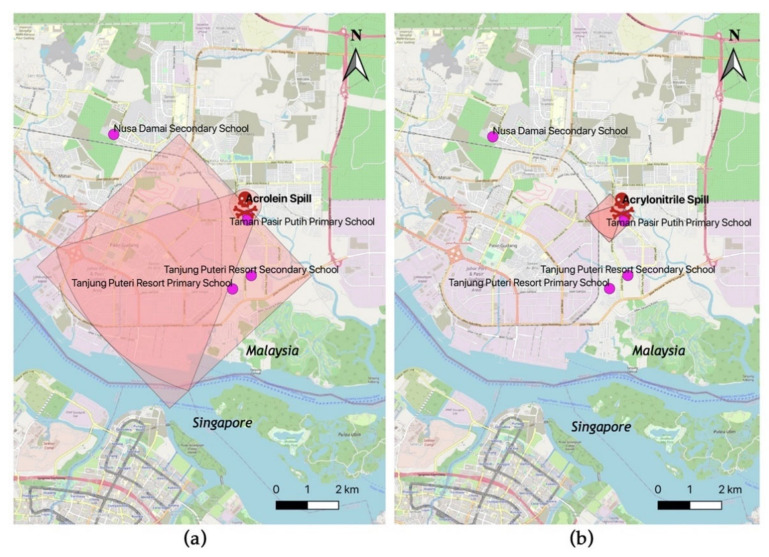
WISER protective distance: (**a**) Protective distance for acrolein, (**b**) protective distance for acrylonitrile.

WISER protective distance analyses showed that the affected area for acrolein and acrylonitrile was within 4.5 and 1 km southwest from the dumping site, respectively (Figure 5).

**Figure 6:**
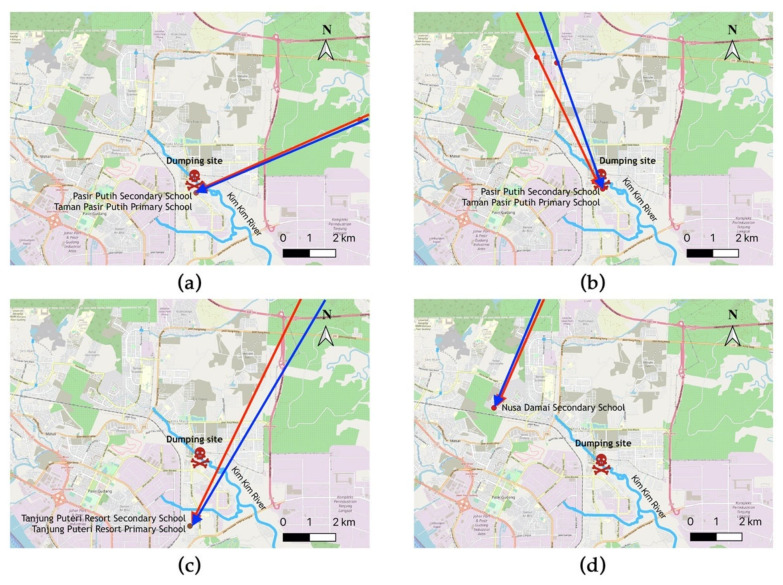
Hybrid Single-Particle Lagrangian Integrated Trajectory (HYSPLIT) model for backward trajectories to the affected schools during the incident of Kim Kim River pollution: (**a**) 7 March 2019; (**b**) 11 March 2019; (**c**) 12 March 2019; and (**d**) 13 June 2019. The red line is the trajectory at 50 m heights and the blue line is the trajectory at 100 m heights.

HYSPLIT backward wind trajectory analyses were carried out to determine the wind direction from the affected school during the incident of Kim Kim River pollution. As shown in Figure 6, the incident occurred on the 7th, 11th, and 12th March 2019, originating from the polluted Kim Kim River. The only incident that did not originate from Kim Kim River was the incident on 13 March 2019 that was probably the result of the sea breeze and land breeze phenomenon [77].

## 4. Discussion

### 4.1. Respiratory Diseases

This study found that most of the children who were taken to hospitals were due to shortness of breath. Studies on environmental exposure largely found a relationship with respiratory symptoms and complications [78,79]. Exposure to benzene has been associated with increased risk of respiratory symptoms in children including wheezing and shortness of breath [80]. Children exposed to 1 μg/m^3^ higher levels of benzene and formaldehyde had a higher odds ratio (OR) of having rhinitis (OR 1.03, 95% confidence interval (CI) 1.01–1.06), exacerbation of bronchial asthma (OR 1.05, 95% CI 1.01–1.10) and dry cough (OR 1.05, 95% CI 1.01–1.10) [78]. Epidemiological studies on the long-term effects due to inhalation of acrolein and acrylamide have not been identified. However, animal and in vitro human airway tissue model studies showing exposure to acrolein and acrylamide were linked to oxidative stress [81,82,83] and inflammation response [84], which led to increased mucus secretions, edema of the bronchial wall, bronchoconstriction, and tissue damage [85]. Hence, this chemical reaction indicated the possibility of children suffering from respiratory symptoms due to exposure to high concentrations of toxic chemicals from the illegal waste dumping site in the Kim Kim River incident.

In terms of the magnitude of disaster, the Pasir Gudang chemical incident was less severe in comparison to the infamous Bhopal disaster that occurred in 1984, whereby 40 tons of methyl isocyanate gas was released following an industrial-related accident [86]. While no human casualties were reported in Pasir Gudang, the Bhopal disaster had resulted in a loss of 10,000 lives within a few days of the occurrence [87]. Another example of a chemical incident that affected respiratory health occurred in 2013 when an ammonia leak incident was reported from a chemical plant located in Rouen, northwest France [88,89]. This chemical leak recorded no fatalities; however, the released chemical had wafted across the English Channel into England, resulting in a transboundary pollution in England. In the situation of the Pasir Gudang incident, there was no transboundary chemical pollution reported.

### 4.2. Cancer

From the result of this study, the risk of developing cancer resulting from the exposure of chemical agents in the Kim-Kim River incident needs further investigation. However, a few studies have investigated the relationship between exposure to benzene and other volatile organic compounds (VOCs) [90,91]. Benzene exposure during childhood was associated with acute lymphocytic leukemia and acute myeloid leukemia of relative risk (RR) 1.0 (95% CI: 0.6–1.7) and RR 1.9 (95% CI: 0.3–11.1) [91]. In general, VOCs can be found around the Pasir Gudang area due to industrial activities such as ship washing activities, and petrochemical and plastic production [92]. As such, poor management of industrial hazardous waste may result in an increase in the risk of developing cancer to the surrounding population.

### 4.3. Mental Health

Exposure to traumatic events either, experiencing or witnessing a harmful situation, poses a threat to an individual’s or population’s mental health. Such events may include, among others, natural disaster, motor vehicle accident, physical injury, and exposure to hazardous material. Approximately 10% of children who were exposed to such events will develop post-traumatic stress disorder (PTSD) [93]. Children with PTSD may experience intrusive memories, avoidance, negative changes in thinking and mood, and changes in physical and emotional reactions. Even though children are generally exposed to the same spectrum of stressors with adults, they are more vulnerable because their emotions and cognition are still immature, they have limited life experience and they lack in coping strategies. Although PTSD was not reported among children following the events in Pasir Gudang, they are at risk of developing PTSD as the onset may develop between 3–12 months post-traumatic event [94].

### 4.4. Limitations

There are several limitations in this study that need to be highlighted. First, this study did not obtain the victims’ biological markers and level of ambient air chemical concentration, due to data confidentiality. Hence, the findings from this research must be interpreted cautiously. Secondly, this study included only children (6–17 years old) from the five affected schools. In reality, there were more children exposed to the hazardous chemical, but due to limitations, it was not possible to measure the whole population of children affected. Therefore, caution should be taken with generalizing the findings to the wider population. Finally, this study only assessed limited numbers of hazardous chemicals involved in the incident. Future research should consider including substantial environmental emissions associated with respiratory diseases among children. The incident in Pasir Gudang may be aggravated by the industrial activities in the area that have now reached saturation level [95].

### 4.5. Policy Implication

The pollution in Pasir Gudang is an example of the failure to learn from past incidents that had occurred in Johor specifically and other places in the country in general [18,19,21,22]. Past experience on similar incidences should be translated into preventive measures as scientific evidence has shown that pollution incidents in the Straits of Johor and Johor’s rivers had been documented and is a repeated occurrence [96,97,98,99,100]. That scientific evidence is important and should be utilized by the relevant enforcement agencies in their effort to improve the quality of services and preventive measures. While the findings of this review may be inconclusive, it indicates that preventive measures are necessary to protect the public’s health from the outcome of an improper hazardous waste disposal. Therefore, this study suggests several recommendations to be considered for future environmental and public health policy makers. First, the health risk assessment framework may be used to identify risks and possible health impacts related to environmental issues. Findings of the health risk assessment may guide policy makers in making environmental policies to improve the situation in the country. Secondly, public health officers should implement the health risk assessment in high-risk areas such as Pasir Gudang to identify vulnerable populations for public health risk management and risk mitigation strategies. Thirdly, all parties should comply with existing environmental laws and regulations, such as the Environmental Quality Act 1974 (Act 127) [101]. Hazardous waste must be disposed of in designated facilities and should not be discarded into the environment such as rivers and landfills. Finally, collaboration between universities, industries and policy makers should be nurtured with the aim of discovering sustainable solutions to complex environmental, health, social, and economic challenges.

### 4.6. Recommendations for Future Work

This study has identified several gaps in the studies of hazardous waste in Malaysia. First, future studies should include children under 5 years old as they are among the vulnerable group in the population. Secondly, the finding of this study has suggested that the chemical incident in Pasir Gudang may cause chronic diseases and mental health issues. Therefore, future cohort research in Pasir Gudang may focus on investigating the incidence of chronic diseases such as cancer and mental health status among children. Lastly, a more comprehensive environmental health assessment should be conducted and documented.

## 5. Conclusions

As a conclusion, this study identified potential health risks to children associated with illegal disposal of hazardous waste in Pasir Gudang, Malaysia. The incident in the Kim Kim River, Pasir Gudang posed a risk of acute respiratory effect. Further investigations on chronic health effects, especially the risk of developing cancer among children, are warranted. The results presented in this study provide important scientific information in promoting health risk assessment to the public health officers and environmental policy makers. The framework of health risk assessment is useful in the process of making informed decisions for risk management and environmental policy improvement. Further, monitoring activities and enforcement actions particularly in developing areas needs to be tightened by the responsible government agencies as strong regulations and good policies alone are not sufficient without the commitment from all stakeholders.

## Figures and Tables

**Table 1:** List of chemicals found at the dumping site and the health effects [63,64].

Chemical	Exposure Route	Symptoms	Lowest Level for Nonlethal Irreversible Damage at 10 to 30 min Airborne Exposure Level	Cancer Risk (IARC)	Inhalation Unit Risk for Cancer
Benzene	Inhalation, skin absorption, ingestion, skin, and/or eye contact	Irritation eyes, skin, and nose; respiratory difficulty; dizziness; headache; nausea; staggered gait; anorexia; lassitude (weakness and exhaustion); dermatitis; bone marrow depression	1100–2000 ppm	Carcinogenic to humans. May cause leukemia	2.2 × 10^−6^ per µg/m^3^
Acrylonitrile	Inhalation, skin absorption, ingestion, skin and/or eye contact	Irritation eyes, skin; asphyxia; headache; sneezing; nausea; vomiting; lassitude (weakness, exhaustion); dizziness; skin vesiculation; scaling dermatitis	3.2–8.6 ppm	Possibly carcinogenic to humans. Increase risk of brain tumor, lung and bowel cancer	6.8 × 10^−5^ per µg/m^3^
Acrolein	Inhalation, ingestion, skin and/or eye contact	Irritation eyes, skin, and mucous membrane; decreased pulmonary function; delayed pulmonary edema; chronic respiratory disease	0.18–0.44 ppm	The agent is not classifiable as to its carcinogenicity to humans	-
Hydrogen Chloride	Inhalation, ingestion, skin and/or eye contact	Irritation nose, throat, and eye; cough; choking; dermatitis; skin burns; contact with refrigerated liquid may cause frostbite	43–100 ppm	The agent is not classifiable as to its carcinogenicity to humans	-
Methane	Inhalation, ingestion, skin and/or eye contact	Breathing difficulties (i.e., suffocation and increased breathing rate); nausea and vomiting; loss of consciousness; weakness; headaches and dizziness; loss of coordination	NA	The agent is probably not carcinogenic to humans	-
Toluene	Inhalation, ingestion, skin and/or eye contact	Headaches; dizziness; loss of consciousness; loss of coordination; sleepiness	760–1400 ppm	Not classifiable as to carcinogenicity to humans	-
Xylene	Inhalation, ingestion, skin and/or eye contact	Irritation of eyes and throat; headaches; dizziness; sleepiness; trembling; lack of coordination	1300–2500 ppm	Not classifiable as to carcinogenicity to humans	-
Ethylbenzene	Inhalation, ingestion, skin and/or eye contact	Irritation of the eyes and/or throat; chest constriction; dizziness	1600–2900 ppm	Possibly carcinogenic to humans	NA
d-limonene	Inhalation, ingestion, skin and/or eye contact	Breathing difficulties; skin irritation	NA	Not classifiable as to carcinogenicity to humans	-

Note: NA = not available.

## Data Availability

No new data were created or analyzed in this study. Data sharing is not applicable to this article.
